# Harmonizing the genetic counselor profession in Europe

**DOI:** 10.1038/s41431-026-02097-8

**Published:** 2026-05-05

**Authors:** Rebecka Pestoff, Christophe Cordier, Donna Darmanin, Katrin Õunap, Christi J. van Asperen

**Affiliations:** 1 Department of Clinical Genetics, Biomedical and Clinical Sciences, Linköping University Hospital, Linköping University, Linköping, Sweden; 2 Clinical Department of Clinical Genetics, Region Östergötland, Linköping, Sweden; 3 Genetic Counselling Unit, Genesupport - Sonic Suisse SA, Lausanne, Switzerland; 4 Department of Pathology, Mater Dei Hospital, Msida, Malta; 5 Department of Genetic and Personalized Medicine, Institute of Clinical Medicine, University of Tartu, Tartu, Estonia; 6 Genetics and Personalized Medicine Clinic, Tartu University Hospital, Tartu, Estonia; 7 Department of Clinical Genetics, Leiden University Medical Center, Leiden, the Netherlands

**Keywords:** Genetic counselling, Genetic services

## Abstract

Genetics in medicine is rapidly becoming integral to European healthcare, yet access to high-quality genetic counseling remains inconsistent. Genetic counseling empowers patients to make informed decisions about genetic testing, improves clinical management, and mitigates psychosocial harm. Despite growing demand, the genetic counselor profession lacks legal recognition, standardized education, and harmonized regulation across European Union (EU) Member States. Current fragmentation, which is evident in separate national laws and variable practices, poses systemic risks, including inequitable care and credentialing barriers. This paper argues that harmonization is essential to ensure ethical, safe, and effective genetic services. We recommend EU-wide legal recognition of genetic counselors, standardized education through EBMG-accredited programs, and investment in workforce development and education. Coordinated action can safeguard individuals’ rights, support professional mobility, and enable responsible integration of genomics into healthcare.

## Introduction

Genetics in medicine is being integrated into both preventive and clinical healthcare across the European Union (EU) to improve patient outcomes and prevention strategies. Patients who undergo genetic investigations and genetic testing or not, need to make informed decisions. Genetic counseling aims to empower patients by providing clear, comprehensive information about the genetic investigation, its significance and implications for themselves and family members through individual support [1, 2], as summarized in Box 1.

Insufficient access to high quality genetic counseling can lead to misunderstandings of diagnostic results, inefficient clinical management, such as delays in early detection, treatment planning, or risk-reducing strategies, higher healthcare costs, and negative psychosocial effects for patients [3]. Thus, we argue that the current expansion of genetic testing in European healthcare must be accompanied by a robust framework of high-quality genetic counseling delivered by appropriately trained professionals.

Box 1 Potential consequences of genetic investigations

- Help to make a diagnosis
- Predict future risks for a disease
- Uncertain clinical implications of DNA test result
- Psychological implications; like issues with mental health, survivor’s guilt, or decisional regret
- Social implications; like issues regarding personal insurance or employment
- Familial implications; regarding relationships and family planning
- Clinical interpretation may change in the future

## Why harmonization matters

Genetics in medicine is creating an increasingly prominent role due to the expanding diagnostic and treatment options. Possibilities for genetic testing touches on many aspects of ethics, psychosocial, medical content, prevention, communication, and fundamental research. Consequently, there are collaborations with numerous departments; the list of Centers of Expertise and publications provide more information on this. The genetic counseling process helps patients understand and adapt to the medical, psychological, and familial implications of genetic disease through risk assessment, education, and support for informed decision-making [1]. The process should be performed by specifically trained Medical Doctors (MDs), who focus on diagnostic issues, and/or non-MD genetic counselors (GCs) who focus more on psychological issues and support. Consequently, the need for qualified GCs to support mainstream genetics grows rapidly in many settings, such as clinical genetics services, specialized clinics, biobank facilities, and population screening programs [3, 4]. MDs trained in Clinical Genetics have EU-wide regulation since 2011 [5]. Yet, the GC profession across Europe remains under-recognized, inconsistently regulated, and unevenly integrated into national healthcare systems [2]. Some countries utilize MDs and GCs, while others allow genetic counseling only by MDs and do not yet use the profession of non-MD GCs [6].

The lack of harmonization at the European level poses a systemic risk. Genetic counseling, like many other health-care service, should be part of routine clinical practice and be provided by qualified professionals [7]. Legal professional recognition serves to protect the public by regulating the safe and competent practice of health care professionals [8]. Patients are entitled to equitable access to quality genetic counseling, and professionals to formal recognition, cross-border mobility, and clearly defined practices. Additionally, consistent standards delivering genetic services in an ethical, safe, and effective manner are needed.

## The current European landscape: growth and fragmentation

The EuroGentest working group undertook a comprehensive review of legislation, policy, and practice across Europe covering the period from 2008 to 2025. A refined literature-search with relevant MeSH-terms in the PubMed database was employed. Identifying more than 300 articles, including several examples of progress, such as increased awareness of the GC role, core competency frameworks, and previous accreditation of education programs [9].

Additionally, gaps were consistently identified across many European countries:

- GC professionals lack legal recognition in most EU countries (only to be found in 2/27 countries);
- The GC role lacks formal regulation, defined expectations, and quality assurance;
- GC educational standards in individual countries are heterogeneous, or non-existent;
- GC workforce capacity is insufficient to meet the growing demand.

A wide disparity in professional integration and legal status exists for GCs in Europe, as shown by the following examples: Only France and Iceland have a legal recognition of GCs [10], and most EU countries maintain a restrictive model [11]. Austria, Belgium, and Germany consider genetic counseling a medical act, only to be realized by MDs [6]. In most Nordic countries, GCs lack legal recognition, but work in teams governed by Medical Geneticists (= MDs), [*personal communication, Nordic Network on Genetic Counseling*]. In the Netherlands, the GC role is filled by Physician Assistants. However, education for this group of health care professionals is not sufficient regarding clinical genetics knowledge [12]. In Estonia there is only one accredited GC (trained in the US) and the reimbursement of genetic counseling service is under consideration (tervisekassa.ee). In Malta, GCs are not recognized, and work under the interim title “Practitioners II (Genomic Care Coordinators)”. This fragmentation is also identified in research, revealing highly inconsistent practices and 18 separate national laws on genetic counseling across the 27 EU Member States. Persistent barriers were identified such as limited genetic literacy among both patients and non-geneticist physicians, and insufficient workforce capacity [3].

The reviewed literature consistently called for harmonized standards, expanded training programmes and ethically grounded, quality genetic counseling services, to allow the advances in genomics to be translated into responsible and equitable clinical practice [7, 13]. The full potential of mainstreaming genetic testing can be reached when complimentary with genetic counseling, and MDs have the medical responsibility.

## The consequences of delayed action

The findings presented here indicate a large variety, which undermines expectations of adequate, quality genetic counseling. The absence of harmonized standards has consequences which include the following:

- Inconsistent access and quality of service [7];
- Psychological harm to patients and families [14, 15];
- Inadequate informed consent and confidentiality breaches [16–18];
- Unnecessary healthcare costs and potential misdiagnosis [3];
- Credentialing barriers for GCs [19];
- Inadequate ethical and legal compliance.

## A European necessity

This working group believes coordinated action is necessary, similarly stated in a recent EU-wide research publication from 2026 [19]. Harmonization is necessary and we recommend European policymakers, professional bodies, and healthcare leaders to take the following actions:

1. Legally recognize GCs as distinct allied-health professionals and establish a European-wide framework for professional recognition.
2. Protect the professional title non-MD “Genetic Counselor” to guarantee accountability, patient safety, and public trust.
3. Standardize education and credentialing by endorsing European Board of Medical Genetics (EBMG)-accredited master’s programs.
4. Invest in workforce development (including genomics training for all healthcare professionals).

Associations such as the European Society of Human Genetics, the EBMG, and EuroGentest are well-positioned to support this effort, which will require collaboration across disciplines, institutions, and borders, as well as coordination, and commitment from the highest political levels. Harmonizing the GC profession in the EU should also coordinate with global efforts, like in Canada [20]. Finally, European countries must prepare to ensure that citizens have access to recognized, competent, and compassionate counseling services by proficiently trained professionals.
